# Elements of Pathological Anatomy

**Published:** 1847-01

**Authors:** 


### Art. III.

*Elements of Pathological Anatomy; illustrated by Coloured Engravings and Two Hundred and Fifty Woodcuts.* By Samuel D. Gross, m.d., Professor of Pathological Anatomy in the Medical Department of the Cincinnati College, &c. Second edition.—*Philadelphia,* 1845. Large 8vo, pp. 822.

We have, in our first Article, noticed an important work of Dr. Gross, published four years since. The present one is of more recent date.

It seems a remarkable fact that the literature of Great Britain does not possess a complete treatise on morbid anatomy. The 'Elements of Pathology' of Mr. Mayo must rather be regarded as a series of notes, than as actually a work, on the subject; besides, throughout its pages the impress of the special surgical pursuits, tendencies, and style of knowledge of the author may be found prevailing to the detriment of the purely medical divisions of his topics. Dr. Hodgkin's treatise, elaborately worked out as far as it goes, is but a fragment; and such, we fear, it is ever likely to remain. Dr. Carswell's fasciculi, models in their way, leave a multitude of subjects either untouched altogether, or but very lightly handled. Besides, no single one of these works contains any account of the microscopic appearances of diseased parts and products. Their publication dates, indeed, before the era of modern or true micrology. Much they contain has been proved absolutely erroneous from the revelations of this, as yet, short-lived era—much, too, requires remodelling and rearranging.

But the deficiency of a work on morbid anatomy, brought up to the level of existing knowledge, is neither traceable to *individual* apathy towards the subject, or inaptitude for the task of its production. There are not a few persons in London at this moment whom we believe both zealous enough and learned enough to accomplish the labour, both with honour to themselves and advantage to their brethren. But these persons seem, naturally enough, disposed to invest their talents in capital more likely to supply a remunerating profit. Experience and inquiry show them that works on morbid anatomy are not prone to return even the outlay necessary for their production. And so long as the subject continues uncared-for in what it is the fashion to style "high places," this must continue to be the case. Few things connected with medical education in this country are more striking than the total indifference on the part of examining boards, as to whether those to whom they grant their diplomas possess even rudimentary knowledge in the facts and principles of this science. Candidates for the licence to treat disease in the human species are required to know minutely the anatomy and physiology of plants, and of the textural changes produced by disease in man it is not cared how ignorant they may be. Youths are peremptorily required to be "thoroughly up" in that which, in nine hundred and ninety-nine of a thousand cases, their subsequent lives will afford them no opportunity of employing, namely, a knowledge of the mode of life of vegetables; they may luxuriate in deficiency, more or less complete, of that on which much of their utility as practitioners of medicine must hereafter depend, namely, a sound knowledge of the nature, sequence, and influence of the anatomical changes preceding, accompanying, or following the development of disease. Hence it is that in no department of medical science does ignorance so generally prevail as in morbid anatomy—a fact of which illustration is too often publicly afforded by evidence given in the courts of law, and (more innocently in respect of consequences) by the "reports" of ambitious tyros in the pages of our medical journals.

As Examining Corporations neglect morbid anatomy—as they do not accord it its due weight and importance as an element of practical education—the majority of medical schools, of course, care not how profoundly ignorant their alumni may remain of its mysteries. The prospectuses of some schools make no mention of the subject at all. Conscious of their deficiency in the reality, the conductors of these establishments make no attempt to produce the semblance of giving instruction in its principles. In the announcements put forth by other schools, instruction in morbid anatomy is advertised by the teacher of some one or two other branches (of which branches, be it remembered, he has scarcely time to expound the facts); and the advertised promise is kept by the chance introduction, some twenty times or so during the course, of allusions, sufficiently profound and mysterious, to John Hunter and coagulable lymph. We appeal to these very teachers themselves, if this be not an unexaggerated statement of their mode of proceeding. We remember, now many years since, meeting the late Dr. Macartney at Paris, to have fallen into conversation with him on the relative conditions of home and foreign medical instruction. The Irish Professor could not contain his indignation at what he called the division and subdivision of subjects at the Parisian School of Medicine. "What outrageous absurdity it is," he exclaimed, "their having distinct professorships of descriptive anatomy, comparative anatomy, surgery, and morbid anatomy! Why, sir," said the doctor, "I have taught every one of these subjects in one course every year for the last twenty years, and I always had time enough, and to spare!!"

There are but two schools, we believe, in London where separate teacherships of morbid anatomy exist; and here, unless our informants have sadly deceived us, the students exhibit their deference for the opinions of the great Examining Corporations—they neglect the teachers. It is, indeed, matter of notoriety that no one has ever succeeded in what is technically termed "making a class" in morbid anatomy in London.

But it may be said that the Society of Apothecaries includes "morbid anatomy" in its curriculum. We admit this, and we give that body full credit for their good intentions: this is not the only instance in which the lowest of the three medical estates has set a good example, which the others have scorned to follow. But good intentions are one thing, and good deeds another. It is well known the desire of the Apothecaries' Company concerning morbid anatomy is virtually a dead letter. Actuated by some evil influence or other, the examining body accepts as evidence of instruction in morbid anatomy the attendance on hospital practice; and thus utterly does away with the intent of those who recognize the importance of serious and systematic study of disease and of morbid changes. Under the present state of things, any pupil who has entered to "hospital practice" obtains his "certificate for morbid anatomy" (though he may never have seen more than a body or two opened) from any one of the hospital physicians!

Despairing then, as we do, for the present at least, of the appearance of a work on morbid anatomy of British manufacture, we have felt no little pleasure in receiving from a transatlantic school the bulky volume of Dr. Gross. This treatise has reached a second edition; and the tribute to its merit thus implied is, within certain limits, not undeserved. Deficient altogether in original facts or views, exhibiting no particular quality of ingenious inference, occasionally mistaken in the statement of opinions and conclusions, incorrect in the allotment of discoveries to their authors, and not unoften displaying want of acquaintance with the existing state of advancement of European science, the volume, nevertheless, is free from errors of importance, and contains a plain and succinct account of the chief facts of morbid anatomy. Dr. Gross, in arranging his materials, has followed the plan of Andral, Carswell, and many others—that of first describing the various morbid states, which occur in the body, by themselves; and then, in connexion with each tissue and organ, dwelling upon the peculiarities which distinguish them therein.

Dr. Gross is a disbeliever in dynamic diseases. "All diseases, I feel confident, will ultimately be found to have 'a local origin and habitation;' and if this opinion shall ever be proved to be true, the whole class of febrile maladies, with its hundred varieties and subdivisions, will cease to have a place in our medical treatises." (p. 26.) We fully coincide in the views here expressed; our admission of dynamic diseases at the present day is merely provisional, and becomes from day to day almost less necessary, in consequence of the discovery of organic causes for numerous diseases presumed formerly to be merely functional.

Dr. Gross holds a doctrine concerning the effusion of serum, the heterodoxy of which is not relieved either by its own plausibility, or by any ingenious argumentation on the part of the author. "Serous effusion," says Dr. Gross, "is the result of inflammation, usually of a very mild grade. That this is true, as a general rule, cannot be doubted; the exceptions, if there be any, are certainly very rare, and have not yet been satisfactorily pointed out." He regards anasarca, for example, of the limbs produced by venous obstruction, as a result of inflammation in the actual part in which the serous effusion occurs. There is, according to him, no such thing as simple mechanical œdema. The sole arguments put in requisition to support his notion are, that (1) the anatomical signs of inflammation may be deficient, or imperceptible, in parts where that process has existed; and (2) when obstruction of a large vein takes place, "the blood is interrupted in its circulation, and congestion of all the vessels, both large and small, is the result. This congestion is not transient, but permanent; and it is scarcely reasonable to presume, judging from our knowledge of the circulation, that this state could exist long without producing an altered condition of the sensibility of the parts affected, attended with more or less redness and effusion of serosity." This is sufficiently hollow reasoning. And, as often happens with propounders of inadmissible notions, the author himself absolutely relinquishes his crotchet (inadvertently, we doubt not) in the very next page; where we find him concluding his inquiry with a statement that serous effusion "is the result invariably of a process *analogous to,* if not strictly identical with, inflammation." (p. 45.) Here the whole position, against which the author contends, is conceded. Everybody knows that there *must, ex naturâ rerum,* be analogy between some of the phenomena occurring in the inflammatory and mechanical processes of serous effusion; but analogy is not identity.

Much of Dr. Gross's volume relating to the subject of inflammation and its effects would be unintelligible, unless upon the understanding that he employs the term organization as synonymous with vascularization. Such employment of the terms would carry us back in pathology more than we should have expected to have been carried back under the conduct of Dr. Gross. The day has passed when the possession of vessels was mistaken for a *sine quâ non* of the organization of an animal substance. Epithelium is surely organized, and the transparent cornea is among the most beautiful specimens of organization the animal body contains; yet vascularity exists in neither the one or the other. Nor does Dr. Gross appear to us happy in his sketch of the mode in which the vessels of solidifying coagulable lymph are produced. The sketch is too sketchy; the real points of anatomical difficulty are scarcely glanced at. But we hold him to be right in his exposure of the pertinacity with which French surgeons refuse to admit the practice of closing amputation and other wounds by first intention. In all probability they will continue to jeopardize (to say the least) the lives of their countrymen by their refusal, until some crafty and most learned Parisian shall have discovered that the practice in question originated in France, and not among "*nos chers confrères d'outremer,*" as we on this side the Straits of Dover are affectionately styled.

Dr. Gross seems particularly fond of detecting and exposing lurking inflammation in conditions where others are agreed that it does not exist. In hemorrhage by transudation, under the influence of mechanical obstruction, he discovers inflammation. At least he doubts whether it does not exist. And upon what evidence? Upon the analogical argument that during menstruation "many women undergo great suffering, labouring under all the symptoms which indicate the presence of inflammation." That the symptoms of ordinary dysmenorrhea are those of an inflammatory state we altogether deny; while, on the other hand, it should have occurred to Dr. Gross that (as is now well known, thanks to the use of the speculum) actual inflammation of the cervix uteri often exists in women who suffer extremely during the menstrual period.

In speaking of "transformations," Dr. Gross discusses as follows a question of some interest. The passage affords a good specimen of his style and manner:

"Is the osseous transformation, when it takes place in the cellular tissue, always preceded by the fibrous and cartilaginous states? Upon this point pathological anatomists are still at variance. If the process be carefully examined, as it occurs in the subserous cellular tissue in different parts of the body, it will be found to involve a series of successive stages corresponding with those that are observed in the fœtal skeleton. The first change which this substance experiences is a diminution of its natural transparency, accompanied with a slight degree of thickening of the part, and a deposition of turbid cream-like matter, which is diffused through its aerolar texture. As the morbid process advances, the part becomes more and more opaque, is rendered flexible and elastic, assumes a grayish colour, and grates under the scalpel. It is now distinctly fibro-cartilaginous; it is next *converted into cartilage*, and finally into bone, the particles of osseous matter being deposited at different points, which gradually augment in diameter; and at length, running into each other, thus completely change the primitive character of the part. The period required for the perfection of each of these changes cannot be determined. In some instances there is reason to believe that it is very short, whilst in others it embraces several months, or some years." (p. 98.)

Dr. Gross has a chapter on "Polypi." We do not believe that in a work on pathological anatomy such a chapter should exist. Polypi possess no special character of *structure* entitling them to consideration apart in a scientific point of view. The word was originally employed to signify a pecularity of *shape* or *form*, and merely this. Polypi are pedunculated masses, composed of structure which is capable of existing in other forms. To us, therefore, it appears that the structure, be it cellular, cellulo-vascular, erectile, or fibrous, &c., should be described generally, and the accidental quality of polypoid shape described as a matter of secondary consequence. The mode of proceeding (the usual one on the part of writers) adopted by Dr. Gross has been productive of the extremely prevalent, and quite as erroneous, notion that the nature of a so-called polypus is peculiar and essential to the fact of its shape. We are still more at variance with Dr. Gross in respect to the views he entertains concerning the "transformations" of polypi. They become carcinomatous frequently, he says; "and, further, the fibrous polypi is by far the most liable to degenerate into malignant [meaning cancerous] disease." (p. 109.) We are well aware that this doctrine has been professed by other persons; but we hold it to be altogether destitute of foundation, as we know it to be destitute of proof. Has Dr. Gross *seen* true fibrous tumours (polypoid or not), wherein a development of cancerous substance had occurred? If so, we sincerely trust he has preserved the specimens. On this side the Atlantic, at least, they would rank amongst the *mirabilia* of morbid anatomy.

Dr. Gross embraces the theory that hydatids owe their origin to inflammation; and the argument he himself adduces in support thereof is in the following wise. In Cincinnati, it appears, the hog is very frequently infected with acephalocysts:

> "Whole droves, consisting of three or four hundred heads, are sometimes thus affected. These animals, most of which are young, are raised in the prairie districts of Ohio, Indiana, and Kentucky, and are literally stuffed with fresh corn for six or eight weeks before they are sent to market. The consequence is, that the portal circle is kept in a state of constant congestion, which finally leads to inflammatory irritation, and the development of acephalocysts in the liver and other viscera." (p. 118.)

Dr. Gross's affection for inflammation, as the prime agent in the production of almost all diseases, leads him occasionally into strange forgetfulness of the principles of logic.

Between the investing cyst of a company of acephalocysts and the animals themselves lies, as is well known, "a soft, pulpy, dirty-looking substance." This intervening substance is supposed by Dr. Gross to be "an *important structure,* designed to assist in the elaboration of a fluid for nourishing the parasite." (p. 119.) It will be admitted that even if this idea be correct (of its correctness no shadow of proof exists), the language in which it is clothed is unfortunate. Dr. Hodgkin, with perhaps greater plausibility, but certainly equal deficiency of proof, regards this same "dirty-looking substance" as an "excrementitious secretion from the hydatid itself." True knowledge is unquestionably not advanced by the promulgation of such gratuitous assumptions.

Dr. Gross's account of tubercle contains two or three opinions and views, which may as well be noticed. For instance, he thus speaks of the gray granulation of the lung:

> "To me the gray granulation seems to be merely a variety of the common tubercle, modified by the action of the affected part, the state of the blood, and the condition of the general health. Serum, lymph, and pus are modified in this manner, and why should not tubercle be? To maintain that the gray granulation is merely a nascent tubercle is, to say the least, not very philosophical. That it is liable to be transformed into the common yellow tubercle is certain; but that death may, and often does, take place before any such change is effected is equally true. The gray granulation may precede the yellow tubercle, or it may be deposited simultaneously with it. Under whatever circumstances it is found, it always contains a disproportionate quantity of animal matter, and is endowed with a much greater power of resisting the influence of such agents as have a tendency to destroy it. It is evidently [?] the product of a more healthy action; it indicates a better state of the solids and fluids; in a word, it is a more plastic organizable substance than common tubercle." (p. 133.)

We freely confess that this train of argument appears to us both obscure and hypothetical. The writer goes on to say that tubercle, being in the first instance liquid, "becomes solid only by the abstraction of its serous particles;" in other words, by a sort of evaporation. Far from this; it is held generally (and we believe with perfect correctness) that the solidification of tubercle depends on the generation of solid particles within its fluid matrix.

Dr. Gross is of opinion that tubercles are always of inflammatory origin. He advances no argument of the slightest novelty in favour of this notion; which, it is needless to inform the readers of this Journal, has, on the evidence of old arguments, been refuted *usque ad nauseam.* But we cannot help commenting on the example here afforded us of the perversity with which exploded errors are sometimes again and again brought back by persons who, in common phrase, should know better. The fact of inflammation, says the author, is proved by the experiments of Cruveilhier and others, who produced "well-characterised tubercles" by dropping mercury into the trachea. Now ought this author not to have known (it is a more unfavorable view to suppose that he concealed this knowledge) that Andral, Gluge, and many others, have shown that the so-called *tubercles* were nothing but small collections of purulent matter? And that, in point of fact, their production throws about as much light on the generation of tubercle as it does upon the means of squaring the circle. Another of Dr. Gross's arguments in favour of the inflammatory origin of tubercle is, that "scirrhus and encephaloid, in fact, all carcinomatous growths, are of inflammatory origin." It is plain that no disputation can be engaged in with a writer who conceives himself justified in making dogmatical assertions, like these, directly at variance with almost universal opinion at the present day.

Writing of muscles, Dr. Gross illustrates his descriptions by woodcuts, copied, without acknowledgment, from British sources. This is, however, a matter of secondary importance,—in one point of view, at least. But it is not a matter of secondary importance, in any point of view, that the rude and totally incorrect drawings of the ultimate texture of muscle, put forward several years ago by Hodgkin and Lister, should, at the present hour, be circulated as representing the state of existing knowledge. Similarly defective is his exposition of the structure of the walls of arteries, which, with anatomists of the old school, he describes as consisting of only three coats. A writer on their morbid anatomy should surely be well acquainted with their structure in the healthy state.

The well-known atheromatous deposit in the coats of arteries Dr. Gross describes under the title of tuberculous; he thinks the disease tuberculous in fact.

"The term which I have here ventured to substitute is, I think, altogether preferable, as it designates at once the true nature [?] of the lesion to which it is applied. Should it be objected that the deposit is not in reality of this description, and, therefore, this name is equally as unphilosophical as the others, I reply that there is nothing to justify such a conclusion, inasmuch as the physical properties of this substance, its mode of secretion, and its final conversion into purulent fluid, all conspire to show its identity with tubercular formations in other parts of the body, especially the bones, testicle, uterus, and seminal vesicle." (p. 230.)

Now this is pathological anatomy gone mad, in our humble opinion. What does Dr. Gross mean by direct observation of the "mode of secretion" of this substance? or, granting that he does know something of the matter, wherein has he found the *peculiarity* in the mode of secretion of this atheromatous matter and of tubercle—the peculiarity (according to his argument) showing that these two products should be separated from others owing their origin to a process of secretion? Again, "its *conversion* into purulent fluid." Who has ever known that the veriest particle of tubercle that ever existed has been, or could be, *converted* into pus? And the looseness of reasoning with which Dr. Gross supports his own notions is rendered more remarkable by his rigid unwillingness to admit the ideas of others (though he adduces no sound objection to them), unless based upon irrefragable proof. Thus he is acquainted with the opinion put forward by Gluge that this change in arteries is really of fatty nature; but, according to this morbid anatomist, "the fact that it contains cholesterine and oily matter does not prove that it is not of a tubercular nature." True enough, in so far as fat and tubercle may be, and occasionally are, associated; but suppose the microscope exhibits *much fat* and *no tubercle*, what then? Will not that prove that the deposit is not tubercular? Now the microscope *has* done this.

"To deny that the articular cartilages are vascular," says Dr. Gross, "as has been done by some, is not less absurd than unphilosophical. Does it follow, because the naked eye is incapable of discerning the produce of vessels, that there must needs exist none? Who has ever demonstrated the vascular structure of the healthy cornea, the arachnoid tunic, or the synovial membranes? No one; and yet that these *organs* [?] are highly organized [vascularized] every pathologist must admit from his own observation." (p. 255.)

Now this passage contains numerous incorrectnesses. In the first place, the opinion that the structures in question are devoid of vessels is not founded alone on examination with the naked eye, but with the microscope; and, besides this, supported by the physiology and pathology of the cartilages. Secondly, no physiologist, that we know of, admits the transparent cornea to be vascular. It has its place among the extra-vascular textures. Dr. Gross appears to have no acquaintance with the physiology and pathology of this class of textures; and hence his description of the diseases of cartilage is a failure. He seems imperfectly acquainted, also, with the history of loose concretions, or "cartilages," of joints,—speaking, as he does, of their being invested with synovial membrane, as of a fact concerning which no doubt may be entertained. Now the truth is, that it is very doubtful whether these bodies are produced outside the synovial cavity in any instance, and certain that in many cases such is not the locality in which they originate. Dropsy of the joints (hydrarthrosis) Dr. Gross sets down as the *invariable* result, when it exists, of chronic inflammation. We do not *know* this idea to be incorrect, but we *believe* it to be so; and unquestionably all the ordinary attendants on inflammation are wanting in many cases of the affection.

Dr. Gross's chapter on diseases of bone is, on the whole, good. He justly understands by the word exostosis "simply a bony excrescence, similar in its structure to the osseous tissue in the normal condition. Nothing, it seems to me, can be more unscientific than the classification of Sir Astley Cooper; who has described under this head some of the most malignant diseases to which the bones are subject." In the justness of this criticism of Sir Astley Cooper's classification we most heartily concur.

The account of diseases of the skin opens with a description of the tegumentary textures in the natural state—a description remarkable for its deficiencies. The microscopical structure of the epidermis is not touched on, and various questions regarding its structure *argued*, which have been long since finally *settled* by the microscope. The whole chapter is bad. Porrigo is described as it might have been described fifty years ago; and not a syllable said of its connexion with the development of vegetable matter of low form. The statement that the vesicles in herpes zoster do not spread beyond the median line is accredited to "Cazenave," apparently under the impression that the statement is of questionable, or, at least, not generally acknowledged, accuracy, and that it originated with the French writer named.

"The arachnoid, like other serous sacs, is liable to effusion of blood." Dr. Gross proceeds to illustrate this statement by an analysis of the papers of some French writers on hemorrhage into the arachnoid cavity in *children,* and appears ignorant of the fact that such hemorrhage is not excessively uncommon in the *adult.* Dr. Carswell's fasciculus, "Hemorrhage," contains every anatomical fact of the least consequence on the subject. That these Frenchmen should know anything of what an English writer may have written on this or any other subject was, of course, not to be expected; but why should Dr. Gross tread in the steps of the ignorant and conceited authors of the Parisian school?

In the first edition of his work, Dr. Gross expressed the opinion that softening of the cerebral tissue is always of inflammatory origin, and "further observation" has, he says, only contributed to confirm this belief.

"Indeed, I cannot see how it is possible to arrive at any other conclusion, especially if we take into consideration the important fact that this lesion (1) occurs at all periods of life, as well as (2) in all parts of the encephalic mass, at one time (3) as an acute, and at another as a chronic affection; (4) that it is often produced by external injury, and (5) by the pressure of certain tumours, or (6) apoplectic effusions; that (7) it is frequently combined with suppuration in other parts of the brain; (8) and, finally, that it occasionally supervenes during the progress of malignant and other fevers." (p. 357.)

We have introduced the figures here for purposes of reference. For the soul of us, we cannot see the force of the arguments from one to four inclusive. The conditions enumerated are not peculiar to diseases of inflammatory origin, and are, therefore, without the kind of significance Dr. Gross would attach to them. Arguments five and six carry little, if any, weight with them. The statement (No. 7) is absolutely incorrect; and (8) the softening during fevers is of a different nature altogether from the "white softening" causing paralysis and contraction. And here, nevertheless, are the arguments which Dr. Gross employs, in proof of the inflammatory nature of the change, in preference to the close anatomical reasoning of Durand-Fardel, whereby the colourless softening is connected, step by step, with a previous condition of red and (by all admitted to be so) indubitably inflammatory softening. This is the more strange, as Dr. Gross makes much use in respect of other points of the volume of Durand-Fardel;* but pathological anatomist though he be, the tendencies of his mind evidently lead him to accord more favour to loose general reasoning than to close deductions from *observed facts.* To the question "does mollescence of the brain ever get well?" he gives as a reply a vague

* We beg to refer the reader to a former Number of this Journal (July, 1843) for a full analysis and estimate of the labours of the French writer.

statement (unintelligible, from its conciseness, it must be to those who have not previously studied the very difficult subject) of the opinions of Cruveilhier and Sims.

Is it not a curious thing to find the author quoting Dr. Stokes of Dublin as an authority for the extreme constancy of the law, that paralysis arising from cerebral disease affects the side of the body opposite to that of the diseased hemisphere? Why any particular authority should be quoted for the fact does not very readily occur to us; still less can we understand why Dr. Stokes should be the individual named. We should have been better pleased to find Dr. Gross attempting to explain upon sure and accurate grounds the cause of the occasional exceptional cases, in which the paralysis exists on the same side of the body as the disease producing it.

Dr. Copland, according to Dr. Gross, has only met with hypertrophy of the brain "*three* times in *several thousand* cases." This is an interesting observation. In order to assign it its entire value, we only require to have some insight *into the number of thousand times* Dr. Copland has opened the cranium and carefully examined the brain! Dr. Gross's description of atrophy of the brain is extraordinarily defective. He makes not the slightest allusion to congenital atrophy (a state of deep interest in all its relations), and leaves his readers without a syllable of information as to the characters of the atrophy of the convolutions (attended with serous effusion) so frequent in lunatics affected with general paralysis.

In turning over the writer's chapter on post-mortem softening of the mucous membrane of the stomach, our attention is attracted by two statements: first, it is not correct to affirm that Louis regards this state as one produced during life, and, "caused by a high state of inflammatory irritation." He *did* once so regard it no doubt, but, in the second edition of his work on typhoid fever (published so far back as 1840), he distinctly renounced his error, and made due acknowledgment of the truth of Dr. Carswell's notions. Secondly (and this is much more important), Dr. Gross states that "if the gastric juice be neutralized by a small quantity of magnesia, no softening, whatever, will happen in the stomach of an animal that has just been killed;" and this statement is of, at the least, excessively doubtful accuracy. We are well aware that the proposition is Dr. Carswell's; but it was the business of Dr. Gross to show that others (Dr. Simpson and Imlach) have found that neutralization of the acid does *not* prevent solution. Admitting this, the admission by no means throws any shadow of doubt on the general truth of the idea of the cadaveric nature of the change,—but it constrains us to look for the solvent agent in some other element of the gastric secretion besides its acid or acids.

Dr. Gross has not properly distinguished the two forms of cicatrization of "simple chronic ulcer" of the stomach,—that form which is attended with puckering of the surrounding mucous membrane, and disappearance of all direct evidence of loss of substance, and the other form, in which a distinct loss of mucous substance is apparent to the eye, while puckering is altogether deficient, and the cicatrix-tissue is a flat fibrous lamella free from corrugation. He commits, too, a practical error in affirming (p. 573) that "the symptoms of carcinomatous disease of the stomach are never so urgent when the lesion is seated near the cardiac extremity, as when it involves the pylorus." Far from this statement being of uniform truth, in some of the most terrible cases (symptomatically considered) of cancerous disease of the stomach on record, the cardiac orifice was the part of the organ implicated. What Dr. Gross *meant* to say was (we descry reason for imagining) probably correct, but he has expressed himself ill.

Dr. Gross treats fully the subject of internal strangulation of the bowels, and we regret that we cannot afford space for an analysis of his chapter on the subject. However, as the merit of his observations consists rather in his excellent arrangement and clear description of the causes of strangulation, than on any actual novelty in his facts or inferences, this is less to be regretted. We like the observation "of the hundred and one *symptoms,* which have been enumerated by authors as indicative of the presence of worms in the alimentary tube; there is only a single one that is of any value, and that is the appearance of these bodies in the evacuations." (p. 633). Perfectly and absolutely true,—and would that, of many of the diseases of the alimentary canal, for which *a hundred and one symptoms* are set down by systematic writers, we had *one* as sure *clinical* indication.

The perusal of Dr. Gross's book leaves, on the whole, a favorable impression on our minds. He may not be a man of first-rate abilities, but he is zealous and honest. On the one hand, he gives no evidence of originality in research or in thought; and on the other, he is unquestionaby deficient in that critical faculty which is essential for the successful sifting of collected materials. But we, nevertheless, willingly admit that he deserves great credit for having executed his labours so well; we receive his book with thanks, and, looking on it as a *transition* work, we recommend it as the most complete and, on the whole, the least defective compilation on the subject in the English language.

---

